# miR‐4286 functions in osteogenesis and angiogenesis via targeting histone deacetylase 3 and alleviates alcohol‐induced bone loss in mice

**DOI:** 10.1111/cpr.13054

**Published:** 2021-05-10

**Authors:** Hongping Yu, Kaiyang Wang, Pei Liu, Pengbo Luo, Daoyu Zhu, Junhui Yin, Qianhao Yang, Yigang Huang, Junjie Gao, Zisheng Ai, Yixuan Chen, Youshui Gao

**Affiliations:** ^1^ Department of Orthopedic Surgery Shanghai Jiao Tong University Affiliated Sixth People’s Hospital Shanghai China; ^2^ The First Affiliated Hospital of Xiamen University Xiamen China; ^3^ Institute of Microsurgery on Extremities Shanghai Jiao Tong University Affiliated Sixth People's Hospital Shanghai China; ^4^ Department of Medical Statistics Tongji University School of Medicine Shanghai China

**Keywords:** alcohol, angiogenesis, HDAC3, miR‐4286, osteogenesis

## Abstract

**Objectives:**

Alcohol consumption is one of the leading factors contributing to premature osteopenia. MicroRNA (miRNA) coordinates a cascade of anabolic and catabolic processes in bone homeostasis and dynamic vascularization. The aim was to investigate the protective role of miR‐4286 in alcohol‐induced bone loss and its mechanism.

**Materials and Methods:**

The effect of miR‐4286 and alcohol on bone mesenchymal stem cells (BMSCs) and human umbilical vein endothelial cells (HUVECs) was explored via multiple in vitro assays, including cell proliferation, QPCR, Western blot, osteogenesis, angiogenesis etc miR‐4286 directly regulated HDAC3 was investigated by luciferase reporter assay, and the function of HDAC3 was also explored in vitro. Moreover, alcohol‐induced bone loss in mice was established to reveal the preventive effect of miR‐4286 by radiographical and histopathological assays.

**Results:**

In vitro, ethanol dramatically inhibited the proliferation and osteogenesis of BMSCs, and substantially impaired the proliferation and vasculogenesis of HUVECs. However, a forced overexpression of miR‐4286 within BMSCs and HUVECs could largely abolish inhibitory effects by alcohol. Furthermore, alcohol‐induced inhibition on osteogenic and vasculogenic functions was mediated by histone deacetylase 3 (HDAC3), and dual‐luciferase reporter assay showed that HDAC3 was the direct binding target of miR‐4286. In vivo, micro‐CT scanning and histology assessment revealed that miR‐4286 could prevent alcohol‐induced bone loss.

**Conclusions:**

We firstly demonstrated that miR‐4286 might function via intimate osteogenesis‐angiogenesis pathway to alleviate alcohol‐induced osteopenia via targeting HDAC3.

## Graphical abstract

miR‐4286 might function via intimate osteogenesis‐angiogenesis pathway to alleviate alcohol‐induced osteopenia via targeting HDAC3. miR‐4286 may be a potential diagnostic and therapeutic target for alcohol‐induced bone loss.

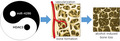

## 1 INTRODUCTION

Osteopenia is a devastating and progressive skeletal disorder with ageing, featured by decreased density of normal mineralized bone with reduced bone strength, which makes individuals more vulnerable to all kinds of fractures, including hip, spine and other skeletal sites.^1^, ^2^ Accumulated previous reports indicated that acute alcohol intoxication suppresses the serum level of osteocalcin (OCN),^3^ and the proliferation of osteoblasts and osteoblastic activity are inhibited by alcohol and metabolite acetaldehyde.^4^ Meanwhile, alcohol remarkably suppressed bone formation evidenced by histomorphology assessment.^5^ Thus, alcohol overuse is considered as a leading risk factor for the secondary osteopenia.^6^, ^7^

Bone mesenchymal stem cells (BMSCs) have a vital role in maintaining bone homeostasis and remodelling by directly differentiating into bone‐forming osteoblasts.^8^ Recent researches indicated that the proliferation and DNA synthesis of BMSCs are significantly inhibited by high‐dose alcohol^9^; and alcohol critically retards mineralization nodes formation and osteogenic differentiation of BMSCs.^10^ Moreover, our previous study showed that both the number and activity of BMSCs are substantially impaired in alcohol‐induced osteopenia.^11^ However, the exact mechanism of alcohol‐induced osteopenia has not been fully elucidated.

Bone formation is a temporal and spatial interaction between vascularization and ossifying tissue, that is angiogenesis‐osteogenesis coupling.^12^, ^13^, ^14^, ^15^ Abundant reports indicated that dynamic vascularization is deeply reduced in osteoporosis.^13^, ^16^, ^17^ Notably, high‐dose alcohol harms neovascularization via inhibiting the expression of angiogenic associated factors.^18^, ^19^ Therefore, the mechanism of chromic heavy alcohol consumption induced osteopenia might depend on inhibiting osteogenesis of BMSCs directly and impairing vascularization to alter bone homeostasis and remodelling indirectly.

MicroRNA (miRNA) is a class of evolutionarily conserved endogenous single‐stranded non‐coding small RNAs (19‐25 nucleotides) and widely expressed in diverse organisms and cells with a multifunctional role in many essential biological processes.^20^ Recent studies demonstrated that miRNAs have crucial regulation on bone formation and remodelling in osteopenia. For instance, miR‐185 depletion and miR‐497/195 cluster prevent osteopenia via promoting osteogenesis and angiogenesis‐osteogenesis coupling.^21^, ^22^ Interestingly, our previous research showed that miR‐136‐3p regulate alcohol‐induced osteopenia via vascularization and bone formation by targeting PTEN.^7^ Histone deacetylases (HDACs) are one of highly conserved enzymes which regulate chromatin structures and signalling events, thereby affect a great deal of activities required for bone formation, regeneration and repair.^23^, ^24^ HDAC3, is highly expressed in bone and cartilage tissues and involved in the physiology of bone homeostasis.^25^, ^26^, ^27^ Notably, sporadic studies indicated that miRNA regulates the bone homeostasis via targeting HDACs, for example, miR‐193b‐3p and miR‐188 regulate BMSCs chondrogenesis, osteogenesis, adipogenesis and metabolism by directly targets HDAC.^26^, ^28^ miR‐4286 was expressed in several species with many physiological functions, like human, mouse and rat.^29^, ^30^, ^31^ In the meantime, based on the result of miRNAs sequencing, we found the expression level of miR‐4286 was significantly decreased. Therefore, miR‐4286 might exert a protective effect in alcohol‐induced osteopenia.

## 2 MATERIALS AND METHODS

### 2.1 Cell culture

The human embryonic kidney 293T (HEK293T) and human BMSCs were purchased from the Cell Bank of the Chinese Academy of Sciences (Shanghai, China). BMSCs and HEK293T cells were cultured in α minimum essential medium and Dulbecco's modified Eagle's medium (Invitrogen). Human umbilical vein endothelial cells (HUVECs) were purchased from Procell (Wuhan, China), and cultured in endothelial cell medium (ECM, ScienCell).

### 2.2 Cell proliferation

The effects of alcohol (100 mmol/L), scrambled miR negative control (miR‐NC) (50 nmol/L, Ruibo, Guangzhou, China) and miR‐4286 mimic (50 nmol/L, Ruibo) on the proliferation of BMSCs and HUVECs were tested by Cell Counting Kit‐8 assay (CCK‐8, Beyotime, Jiangsu, China). The dose of ethanol was according to the previous work.^32^ In brief, cells were seeded in 96‐well plate at 5 × 10^3^ per well in 100 μL culture medium. 10 μL CCK‐8 solution and 90 μL medium were added and incubated in 37℃ for 2 hours. The absorbance values were measured.

### 2.3 Osteogenesis assay

Bone mesenchymal stem cells were cultured with alcohol (100 mmol/L), miR‐NC (50 nmol/L), miR‐4286 (50 nmol/L), the HDAC3 antagonist Nexturastat A (Nex, 5 μmol/L, Selleck, Shanghai, Chin) for specific time.^33^ In brief, 2 × 10^5^ BMSCs were seeded in 6‐well plates. After 80% confluence, osteogenic medium (Cyagen, Suzhou, China) was added and refreshed. ALP activity was detected at days 7 and 14. Alizarin red staining (ARS) and ALP staining were obtained.

### 2.4 Wound healing assay

Human umbilical vein endothelial cells were seeded into a 6‐well culture plate. Then, the confluent cells were wounded by scratching the monolayer with a sterile pipette tip. Cells were then cultured with culture medium under different treatments. Images were captured, and the rates of wound healing were measured by Image J.

### 2.5 Transwell assay

2 × 10^4^ cells per well were seeded in the upper chambers of a 24‐well transwell plate and 700 μL of conditional medium were added to the lower chambers. Then cells were fixed, erased cells on the upper surface of the membrane and the cells on the lower surface were stained with crystal violet. Finally, total cells of 6 random lower surfaces were chosen and counted in microscope.

### 2.6 Tube formation assay

In brief, 50 μL/well Matrigel (BD Bioscience) was added to the 96‐well plate. Then, 4 × 10^4^ HUVECs/well were seeded on the surface of Matrigel after gelatinization in 37℃ for 30 minutes. The tube formation images were captured 6 hours later by a LEICA phase‐contrast microscope, and the number of complete capillaries and nodes of each hole was counted.

### 2.7 Quantitative real‐time polymerase chain reaction (QPCR)

Bone mesenchymal stem cells or HUVECs were cultured in 6‐well plates for 48 hours and then total mRNA or miRNA was extracted according to the manufacturer's protocols (EZBioscience). As for the extraction of mRNA or miRNA in distal femurs, the bone marrow was flushed out and the bone tissue was grinded with liquid nitrogen before the use of TRIzol. Reverse transcriptase reactions had the purified RNA and 50 nmol/L RT primer. QPCR was performed by a SYBR Premix Ex Taq protocol (EZBioscience) on an MX3005P system. GAPDH or U6 was set as the internal gene of RNA expression. The primers were listed in Table S1.

### 2.8 Western blot

In brief, BMSCs and HUVECs were lysed with RIPA lysis buffer (1 mmol/L PMSF, Beyotime). As for the extraction of protein in distal femurs, the bone marrow was flushed out and distal femurs were grinded with liquid nitrogen before the addition of RIPA lysis buffer. The protein concentrations were detected and 30 μg protein was electrophoresed and transferred to PVDF membranes. Membranes were blocked and incubated with the primary antibodies against OCN, GAPDH, HDAC3 (1:1000, CST). Then, the membranes were washed and incubated with an HRP‐conjugated second antibody (1:5000). Finally, the membranes reacted and signals were quantified.

### 2.9 Enzyme‐linked immunosorbent assay

Human umbilical vein endothelial cells were cultured in 6‐well plates. After 80% confluence, cells were cultured with serum‐free medium for 48 hours for detecting the level of VEGF. Then the supernatant liquor was diluted for analysis of VEGF content by ELISA (Neobioscience, Shenzhen, China). The absorbance values were detected at 450 nm and used to calculate the VEGF content according to the standard curve.

### 2.10 Immunofluorescent staining

Bone mesenchymal stem cells were seeded on 35 mm confocal dishes with conditional medium. Later, cells were washed and fixed, permeabilized and incubated with antibodies against for COL I and OCN (1;100, CST). Cells were washed and incubated with the Alexa FluorTM 488 secondary antibodies (1:500). Subsequently, cell nucleus and skeletons were stained with DAPI and phalloidin. Images were captured by a LEICA microscope.

### 2.11 Animal experiment

Thirty 8‐week‐old male C57BL/6 mice were used for animal experiment with the approval from the Animal Research Committee at Shanghai Sixth People's Hospital. All mice were randomly and equally divided into three groups: (1) miR‐NC group, (2) alcohol + miR‐NC group and (3) alcohol + miR‐4286 group. In order to induce osteopenia after one‐week adaption, all mice were fed with alcohol in drinking water (5%‐30% by progressive and final concentration is 30%) for 6 weeks according to the previous report.^34^ The drinking water was refreshed every 2 days in order to maintain the concentration of alcohol. All mice were free access to the diets. For animals receiving miR treatment, the mice were injected intravenously with agomir‐NC (80 mg/kg, Ruibo) or agomir‐4286 (80 mg/kg, Ruibo) according to the previous reports.^21^ In brief, injection was performed at the first and the fourth week, respectively.

### 2.12 Micro‐CT scanning and histomorphometry

Mice were sacrificed under general anaesthesia at the end of the 6th week. Femurs were obtained and fixed and scanned with a 9‐micron voxel size micro‐CT scanner (Skyscan 1176, Kontich, Belgium). The images were managed and reconstructed. Bone parameters were calculated from the reconstructed images, including bone mineral density (BMD), trabecular thickness (Tb.Th), trabecular number (Tb.N) and trabecular bone volume fraction (BV/TV).

After micro‐CT scanning, femurs were decalcified and embedded in paraffin or in OCT compound. The samples were cut into 5‐μm‐thick sections and stained with haematoxylin and eosin (HE), Masson trichrome, Safranin O/Fast Green solutions, Immunofluorescent staining (EMCN, CD31, 1:100, CST), immunohistochemical staining (COL I and OCN,1:100, CST).

### 2.13 Dual‐luciferase reporter assay

Plasmid containing HDAC3 wild or mutant 3′‐UTR was fused to the 3′ end of psiCHECK2 luciferase vector (Promega, Madison, WI). The wild type and mutant fragments of the HDAC3 3′‐UTR involving the predicted miR‐4286 target sites (Positions 493‐498) were directly synthesized (Obio, Shanghai, China). The HEK293T cells were transfected with the plasmids using Lipofectamine 3000 reagents (Life Technologies) with miR‐NC or miR‐4286 mimic. Cells were collected after 48 hours transfection, the activities of Renilla and firefly luciferases were detected by a dual‐luciferase reporter assay kit (Promega). Relative luciferase activities were calculated via normalizing Renilla luciferase activity to the firefly luciferase activity.

### 2.14 Statistical analysis

All data were presented as means ± standard error of mean (SEM). The differences between groups were determined by Student's *t* test or one‐way ANOVA with Bonferroni's correction in SPSS 18 (IBM, Armonk, NY). ^*^ *P* < .05, ^**^ *P* < .01 and ^***^ *P* < .001 were of statistical significance.

## 3 RESULTS

### 3.1 miR‐4286 rescued the inhibitory effects of alcohol on the proliferation and osteogenesis of BMSCs

**FIGURE 1:**
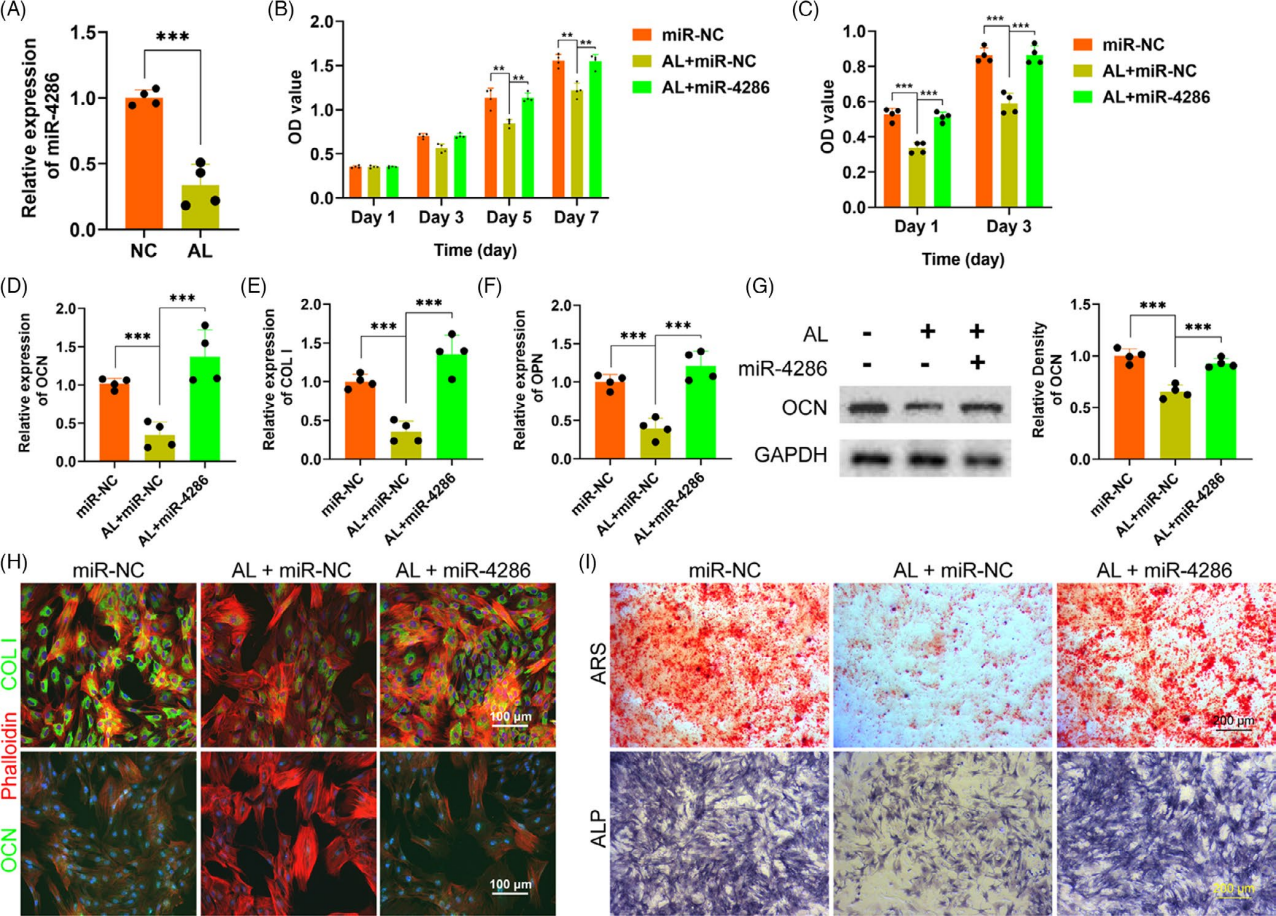
miR‐4286 rescued alcohol‐induced inhibitory effects on BMSCs. A, The expression level of miR‐4286 was remarkedly decreased. B, The CCK‐8 assay was used to evaluated the proliferation of BMSCs. C, The ALP activity treated with alcohol and miR‐4286 on BMSCs. D‐F, The gene level of COL I, OCN and OPN treated with alcohol and miR‐4286 on BMSCs. G, The protein level of OCN was detected by alcohol and/or miR‐4286 on BMSCs. H, IF of COL I (green) and OCN (green). Cytoskeletons were stained with phalloidine (red), and the nucleus was stained with DAPI (blue). I, The ARS and ALP staining of BMSCs

The expression of miR‐4286 in BMSCs administrated with alcohol was detected by QPCR. As shown in Figure 1A, miR‐4286 was remarkedly decreased in 100 mmol/L alcohol as compared with the control (NC). Next, CCK‐8 assay was performed to investigate the effect of alcohol and miR‐4286 on the proliferation of BMSCs. 100 mmol/L alcohol significantly inhibited BMSCs proliferation at day 5 and 7, while 50 nmol/L miR‐4286 could rescue the inhibitory effect of alcohol (Figure 1B). Then we studied the effect of alcohol and miR‐4286 on the osteogenesis of BMSCs. The ALP activity of BMSCs was distinctly inhibited by alcohol, while co‐treatment with miR‐4286 could largely abolish the inhibition (Figure 1C). The gene expressions of the osteogenic‐associated markers COL I, OCN and OPN were critically reduced after alcohol administration, however, these inhibitory effects of alcohol were rescued by miR‐4286 (Figure 1D‐F). We also investigated the protein expression of OCN, which was significantly impaired by alcohol, however, miR‐4286 could reverse the inhibition of alcohol (Figure 1G). Immunofluorescent staining was performed to directly visualize and quantify the changes of osteogenic‐associated markers within BMSCs. The results indicated that alcohol distinctly decreased the protein level of COL I and OCN for 48 hours, but co‐treatment with miR‐4286 obviously rescued the inhibition of alcohol on BMSCs (Figure 1H). Finally, ARS and ALP staining were conducted to observe the osteogenic differentiation of BMSCs. Alcohol obviously inhibited the mineralization node formation and ALP activity in BMSCs, while miR‐4286 could reverse the inhibition by alcohol (Figure 1I).

### 3.2 miR‐4286 rescued the inhibitory effects of alcohol on vascularization in vitro

**FIGURE 2:**
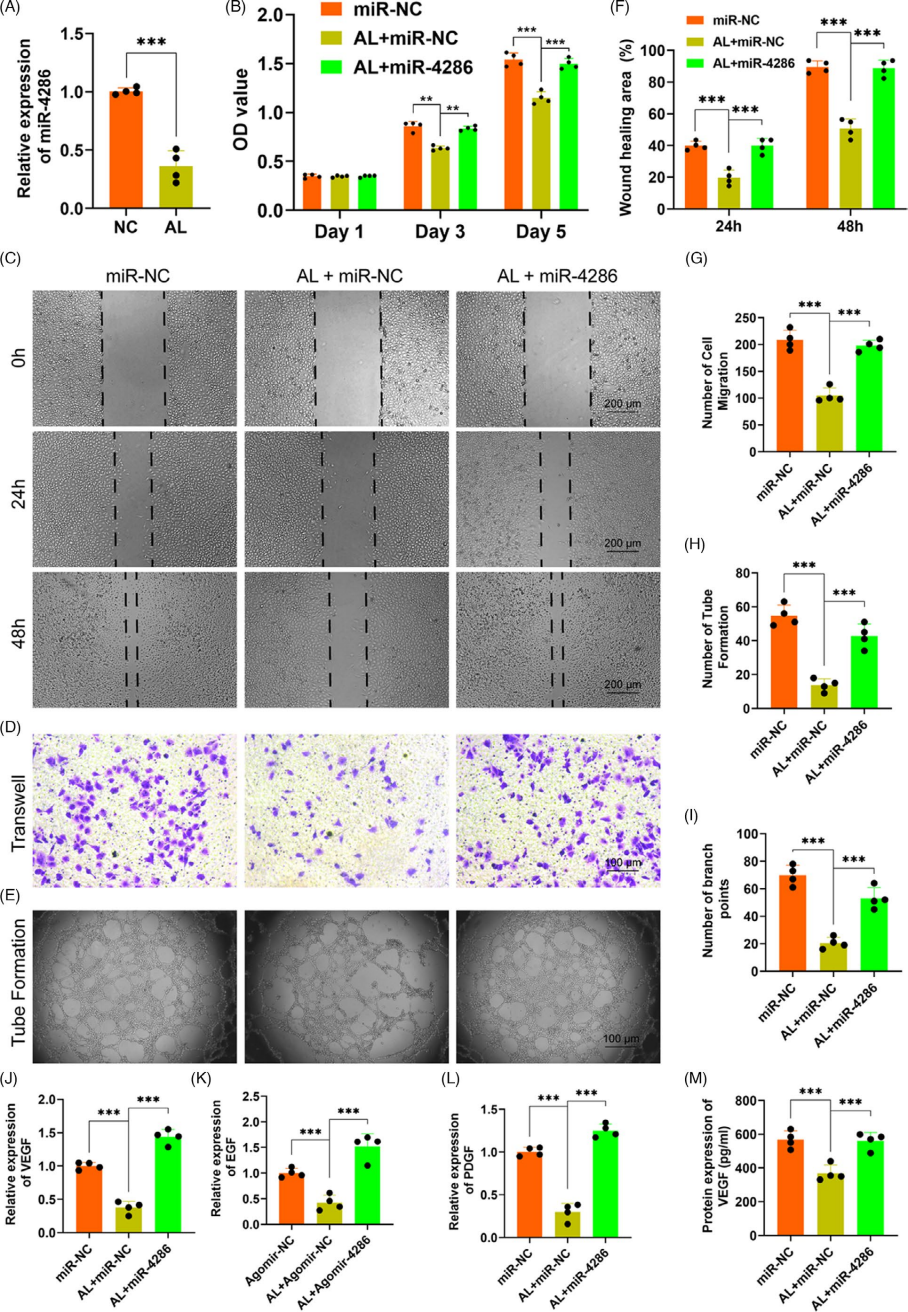
miR‐4286 rescued alcohol‐induced inhibitory effects on HUVECs. A, The expression level of miR‐4286 on HUVECs. B, The CCK‐8 assay was used to evaluated the proliferation of HUVECs. C‐E, The representative images of wound healing, transwell and tube formation of HUVECs. F‐I, The statistical results of wound healing, transwell and tube formation of HUVECs. J‐L, The mRNA levels of VEGF, EGF and PDGF in HUVECs. M, The protein levels of VEGF in HUVECs

The expression of miR‐4286 within HUVECs treated with alcohol was investigated by QPCR. The results showed that miR‐4286 was dramatically reduced by alcohol within HUVECs as compared to the NC (Figure 2A). Then, the effects of alcohol and miR‐4286 on the proliferation of HUVECs were investigated by CCK‐8 assay. Our data demonstrated that alcohol distinctly inhibited the proliferation of HUVECs at day 3 and 5, while miR‐4286 could rescue the inhibitory effect of alcohol on HUVECs (Figure 2B). Next, wound healing and transwell assay were performed to investigate the effects of alcohol and miR‐4286 on the migration capacity of HUVECs. The experimental results indicated that the wound healing area and the number of migrations of HUVECs were distinctly inhibited by alcohol, while miR‐4286 could rescue the inhibitory effects of alcohol on migration of HUVECs (Figure 2C,D and F,G). Subsequently, the effects of alcohol and miR‐4286 on the vasculogenesis of HUVECs were investigated by tube formation assay. As shown in Figure 2E and H,I, there were much fewer loop structures and number of branch points in alcohol‐treated HUVECs, while miR‐4286 could rescue the inhibition of alcohol on tube formation of HUVECs. The QPCR results indicated that the angiogenic‐associated gene expressions of VEGF, EGF and PDGF were significantly impaired within HUVECs by alcohol, while miR‐4286 could remarkably reverse the inhibitory effects caused by alcohol (Figure 2J‐L). We also measured the level of VEGF by ELISA. VEGF was significantly reduced by alcohol, but significantly rescued by miR‐4286 (Figure 2M).

### 3.3 Alcohol inhibited the osteogenesis of BMSCs via upregulating HDAC3

**FIGURE 3:**
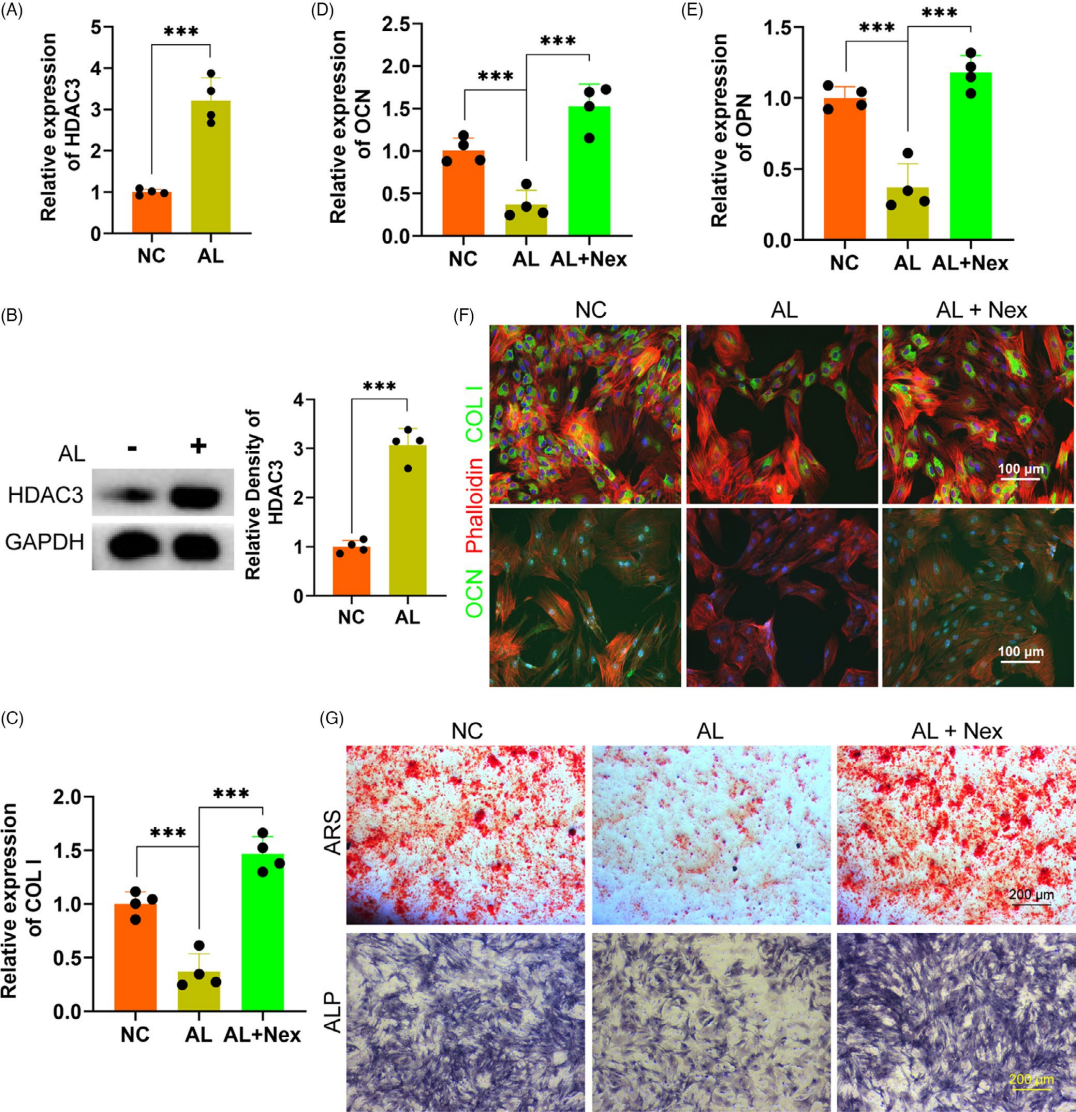
Alcohol inhibited the osteogenesis of BMSCs via HDAC3. A‐B, The expression level of HDAC3 in BMSCs. C‐E, The gene level of COL I, OCN and OPN. F, IF of COL I (green) and OCN (green). Cytoskeletons were stained with phalloidine (red), and the nucleus was stained with DAPI (blue). G, The ARS and ALP staining of BMSCs

In order to explore the exact mechanism of the inhibition of alcohol on the osteogenic differentiation of BMSCs, we detected the potential role of HDAC3 in alcohol‐treated BMSCs. The gene level of HADC3 of BMSCs was remarkably upregulated by alcohol treatment (Figure 3A). The protein level of HDAC3 was further measured by Western blot, showing alcohol obviously increased HDAC3 in BMSCs treated with alcohol (Figure 3B). Subsequently, the HDAC3 antagonist Nex was used to investigate the exact role of HDAC3. The results indicated that the gene expressions of the osteogenic‐associated markers COL I, OCN and OPN were obviously decreased by alcohol within BMSCs, while these inhibitory effects of alcohol were completely reversed by Nex (Figure 3C‐E). Immunofluorescent staining demonstrated that Nex treatment significantly rescued the decreased protein of COL I and OCN (Figure 3F). Finally, ARS and ALP staining showed that co‐treatment with Nex could against the inhibition of alcohol on mineralization node formation and ALP activity in BMSCs (Figure 3G).

### 3.4 Alcohol inhibited the proliferation, migration and tube formation of HUVECs via enhancing HDAC3

**FIGURE 4:**
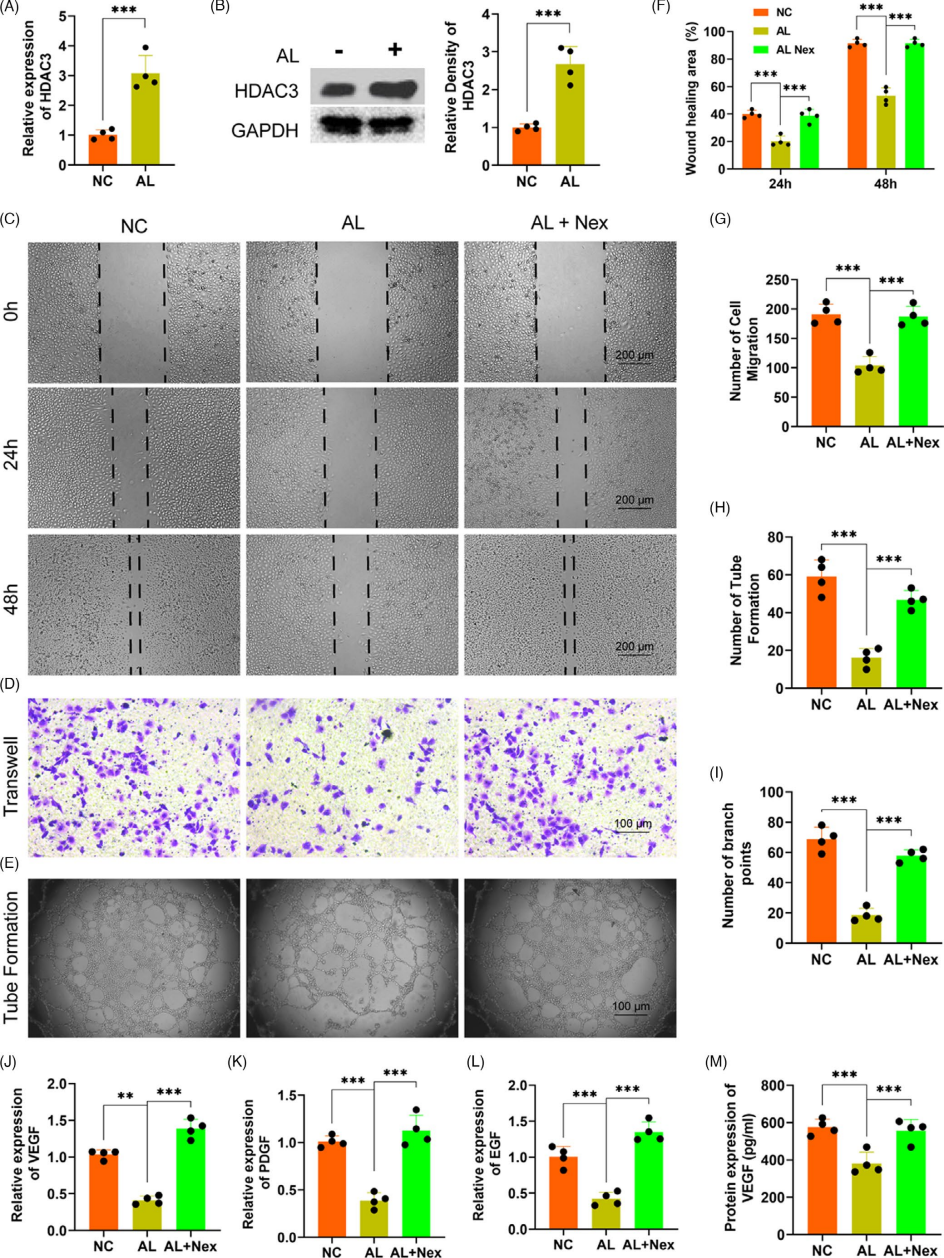
Alcohol inhibited the migration and tube formation of HUVECs via HDAC3. A‐B, The expression level of HDAC3 on HUVECs. C‐E, The representative images of wound healing, transwell and tube formation of HUVECs. F‐I, The statistical results of wound healing, transwell and tube formation of HUVECs. J‐L, The mRNA levels of VEGF, EGF and PDGF in HUVECs. M, The protein levels of VEGF in HUVECs

We detected the potential role of HDAC3 in alcohol‐treated HUVECs to investigate the exact mechanism of the inhibition of alcohol on proliferation, migration, tube formation of HUVECs. Firstly, the gene expression of HADC3 was distinctly upregulated by alcohol as compared to the NC treatment (Figure 4A). The protein level of HDAC3 was also obviously increased by alcohol within HUVECs (Figure 4B). Secondly, the HDAC3 antagonist Nex could remarkably rescue the alcohol‐induced inhibitory effect on wound healing and HUVECs migration as evidenced by wound healing and transwell assay (Figure 4C,D and F,G). Thirdly, alcohol‐induced inhibition on loop structure formation and branch points by HUVECs were obviously rescued by Nex administration (Figure 4E and H,I). Finally, the QPCR results indicated that Nex could significantly against the alcohol‐induced detrimental effect on the gene expressions of the angiogenic‐associated markers VEGF, EGF and PDGF (Figure 4J‐L). ELISA further demonstrated Nex could distinctly rescue the alcohol‐induced downregulation of VEGF by HUVECs (Figure 4M).

### 3.5 miR‐4286 targeted HDAC3 in the dual function of vascularization and bone formation

**FIGURE 5:**
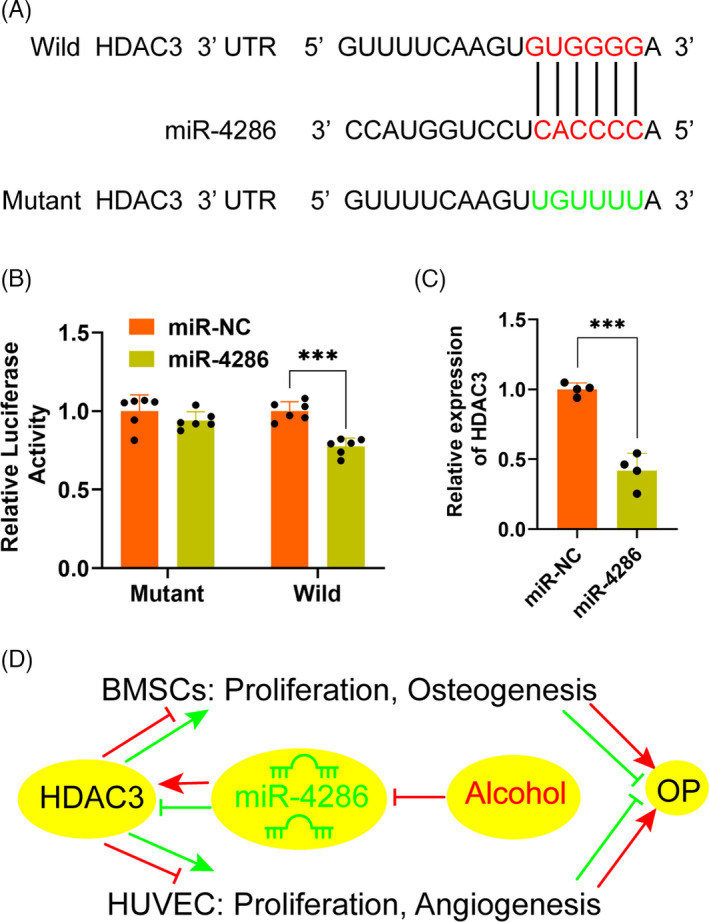
HDAC3 is the direct target of miR‐4286 (A) Construction of the wild HDAC3 3′‐UTR or mutant HDAC3 3′‐UTR plasmid. The potential target sites of HDAC3 3′‐UTR for miR‐4286 were shown. B, Normalized luciferase activity of the vectors after miR‐4286 overexpression in HEK293T cells. C, The level of HDAC3 was remarkedly downregulated after miR‐4286 overexpression in HEK293T cells. D, Schematic representation of the regulating effect of the miR‐4286 on alcohol‐induce osteoporosis in mouse model

In order to elucidate the molecular mechanism underlying HDAC3 and miR‐4286, the sequence of the 3′‐UTR of HDAC3 mRNA was analysed by TargetScan (http://www.targetscan.org). Results revealed a potential miR‐4286 binding site in the 3′‐UTR of HDAC3 (Figure 5A). Luciferase reporter assay with wild and mutant 3′‐UTR HDAC3 was performed to investigate whether miR‐4286 directly regulated HDAC3 production. miR‐NC and miR‐4286 mimic was, respectively, transfected into HEK293T cells with luciferase reporter plasmid. The luciferase activity of wild 3′‐UTR of HDAC3 was distinctly downregulated by miR‐4286 mimic, while the luciferase activity of mutant 3′‐UTR of HDAC3 was slightly depressed (Figure 5B). Moreover, it was demonstrated that the level of HDAC3 was significantly reduced in HEK293T cells transfected with miR‐4286 mimic, as compared to miR‐NC mimic transfection (Figure 5C). Taken together, these results demonstrated that miR‐4286 might prevent alcohol‐induced osteopenia via targeting HDAC3 by dual regulation of osteogenesis and vascularization (Figure 5D).

### 3.6 miR‐4286 attenuated alcohol‐induced bone loss in mice

**FIGURE 6:**
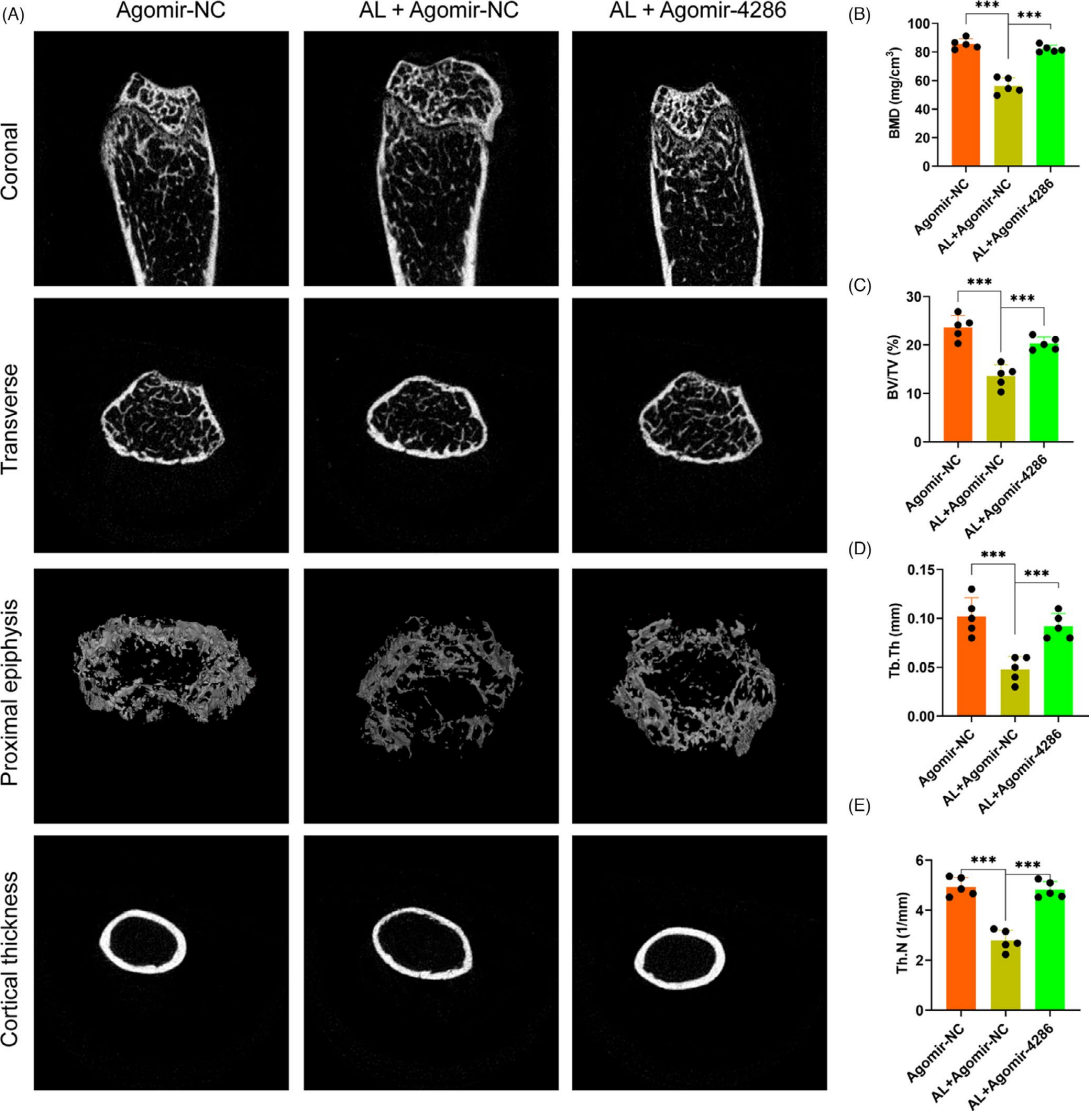
Micro‐CT scanning and analysis of the distal femur of mice. A, Micro‐CT scanning and 3D reconstruction images of the distal femurs. B‐E, BMD, Tb.N, BV/TV and Tb.Th were calculated based on the reconstructed CT images

To investigate the role of miR‐4286 in the progression of alcohol‐induced osteopenia, C57BL mice were subjected to either agomir‐NC or agomir‐4286. Micro‐CT scanning was performed to analyse the bone parameters and microstructure destruction of the femur epiphyses of the mice. The 3D reconstruction images showed that specimen from the agomir‐NC + alcohol group displayed a decreased trabecular number and volume, reduced epiphyseal plate and much thinner bone cortex, as compared to the agomir‐NC group (Figure 6A). However, agomir‐4286 could largely reverse the alcohol‐induced osteopenia in mice (Figure 6A). Moreover, the deleterious effect of alcohol and the protective effect of miR‐4286 were further investigated via quantitative analysis of the micro‐CT data. Animals from the agomir‐NC + alcohol group displayed significantly decreased bone parameters, including BMD, Tb.N, BV/TV and Tb.Th, as compared to the agomir‐NC group, while agomir‐4286 significantly restored these bone parameters (Figure 6B‐E).

**FIGURE 7:**
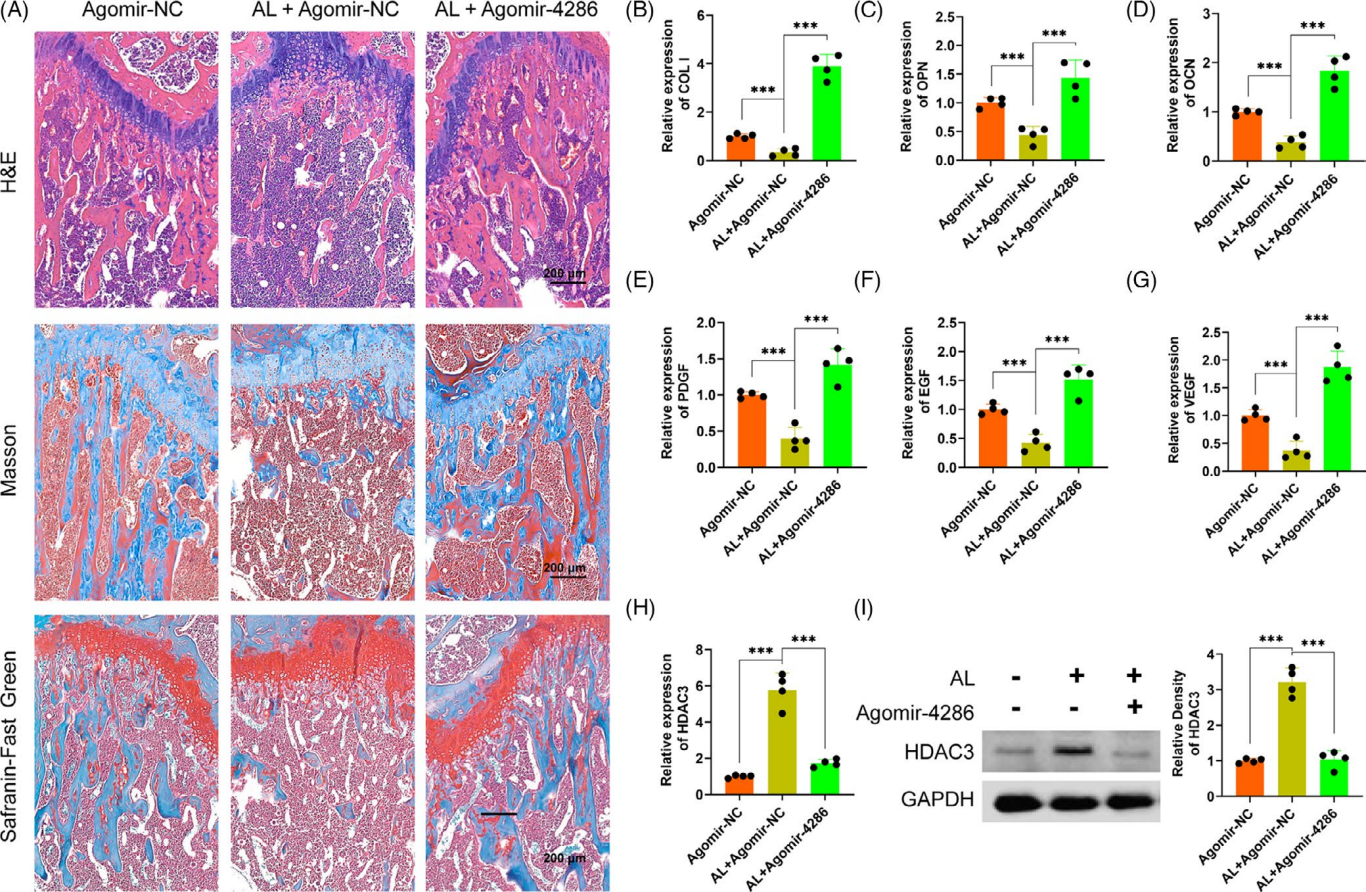
Alcohol‐induced osteoporosis in the mice model was alleviated by miR‐4286. A, Representative images of H&E, Masson's trichrome and Safranin O‐Fast Green staining. B‐G, The gene level of osteogenesis and angiogenesis treated with alcohol and agomir‐4286 in the distal femurs of mice model. H‐I, The gene and protein level HDAC3 treated with alcohol and agomir‐4286 in the distal femurs of mice model

**FIGURE 8:**
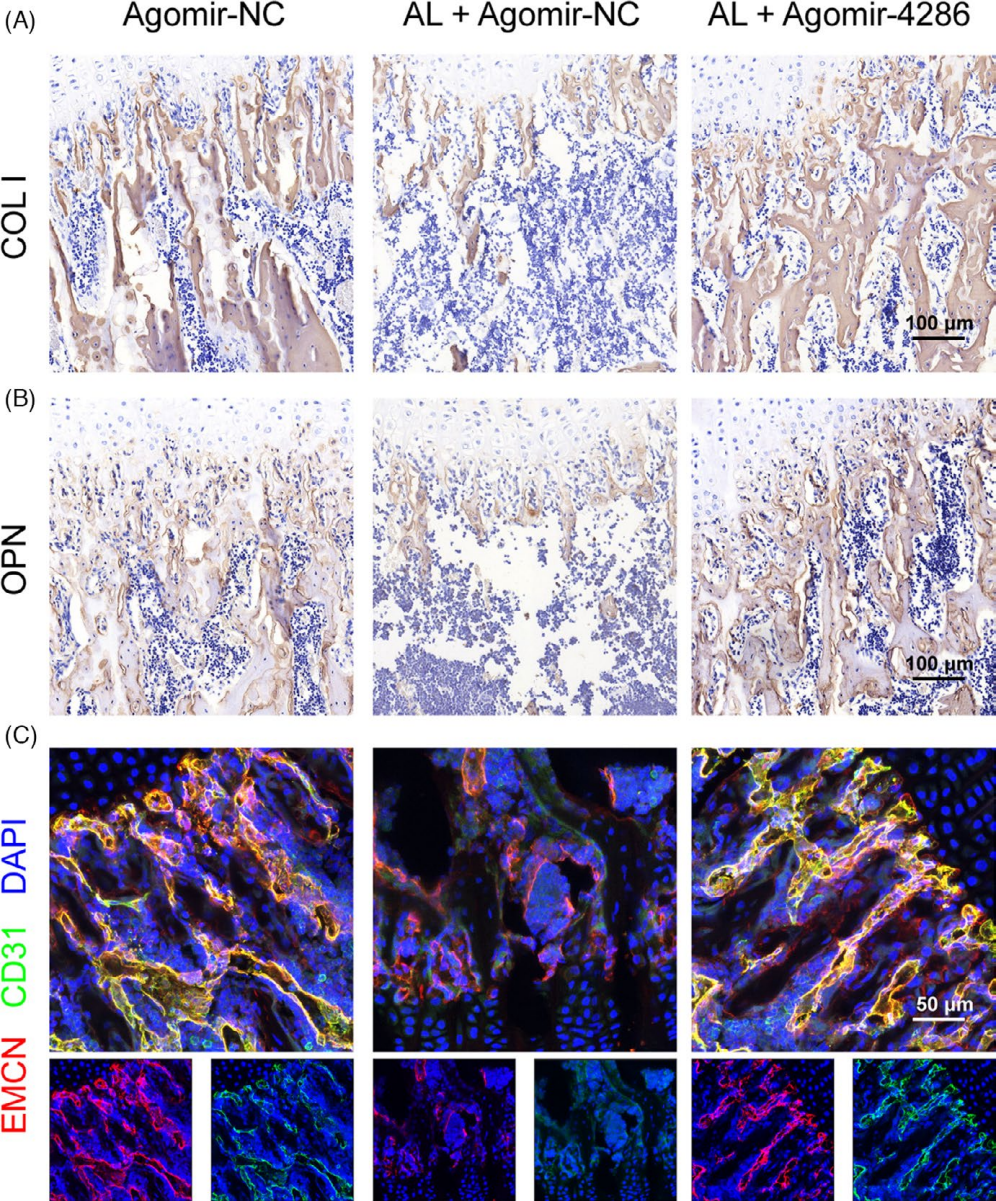
Alcohol impaired new bone formation and vascularization in mice and HDAC3 was the direct target of miR‐4286. A‐B, Immunohistochemical staining of COL I and OPN in the distal femur of mice. C, Immunofluorescent staining of EMCN and CD31 in the distal femur of mice, EMCN (red), CD31 (green), DAPI (blue)

The alcohol‐induced osteopenia in mice was further confirmed by histological staining. As compared with the agomir‐NC group, the agomir‐NC + alcohol group had obviously weaker collagen staining and sparser trabecular bone area, reflected by Masson's trichrome and Safranin O‐Fast Green staining (Figure 7A). However, there were significantly greater collagen staining and denser trabecular bone area in the agomir‐4286 group (Figure 7A). Furthermore, the QPCR results indicated much lower osteogenic‐associated markers expression of COL I, OCN and OPN in the agomir‐NC + alcohol group than the agomir‐4286 group in the femurs of mice (Figure 7B‐D). The angiogenic‐associated markers of VEGF, EGF and PDGF were also much lower in the agomir‐NC + alcohol group (Figure 7E‐G). Notably, the gene and protein expression levels of HADC3 was distinctly upregulated in the agomir‐NC + alcohol group than the agomir‐NC group in the femurs of mice, while miR‐4286 could obviously downregulate the alcohol‐induced upregulation of HDAC3 in femurs of mice (Figure 7H,I). Finally, we performed the relevant osteogenic and vascular staining in vivo to investigate the effect of alcohol and miR‐4286 on mice. The alcohol group had obviously decreased positive staining of COL I and OPN which indicated significantly reduced osteogenic activity proofed by immunohistochemical staining of COL I and OPN in the distal femur of mice, while miR‐4286 treatment could reverse alcohol‐induced reduced osteogenic activity (Figure 8A,B). The immunofluorescent staining of EMCN and CD31 indicated that the alcohol group exert reduced number of vessels evidenced by significant decreased staining density of EMCN and CD31 in the distal femur of mice; on the contrary, the inhibitory effect of alcohol on vasculogenesis in mice was reversed by co‐treatment with miR‐4286 (Figure 8C).

## 4 DISCUSSION

A substantial number of premature osteopenia in men have a history of alcohol overuse.^35^ Chronic heavy alcohol consumption reduces bone mineral density, cortical and cancellous architecture, resulting in the imbalance of bone formation and remodelling. Notably, the pathophysiology of osteopenia is the disordered remodelling between bone formation by osteoblasts and bone resorption by activation of osteoclasts.^36^ BMSCs are the precursor cells of osteoblasts and can differentiate into osteoblasts. Recent researches indicated that osteopenia is largely due to the impaired osteogenic differentiation of BMSCs.^37^, ^38^, ^39^ Therefore, BMSCs have a vital role in the occurrence and development of progressive bone loss.

miRNAs exert a crucial regulation on bone formation and remodelling in the occurrence and development of osteopenia via regulating osteogenesis of BMSCs or angiogenesis.^20^ miR‐185 and miR‐497/195 could regulate bone homeostasis via promoting osteogenesis and regulating angiogenesis‐osteogenesis coupling, respectively.^21^, ^22^ The current study, for the first time, demonstrated that miR‐4286 can against alcohol‐induced osteopenia by the dual regulation of osteogenesis as well as angiogenesis in mice, via modulating the expression of HDAC3. Our experimental results showed that miR‐4286 protected alcohol‐induced osteopenia via targeting HDAC3 based on the following solid evidence. First, the TargetScan predicted that the 3′‐UTR of HDAC3 had a potential miR‐4286 binding site. Second, miR‐4286 overexpression significantly inhibited the activity of wild HDAC3 but not mutant HDAC3. Finally, the level of HDAC3 was critically reduced after miR‐4286 overexpression in HEK293T cells and alcohol‐fed mice.

The latest studies have shown that HDAC3 exerts a vital role in the physiology of bone homeostasis. The level of HDAC3 was remarkably decreased during the odontoblast differentiation^40^; and the inhibition of HDAC3 activity promoted the osteogenesis of human periodontal ligament cells.^41^ In the downstream, HDAC3 interacts with Runx2 to repress the OCN promoter and regulate osteogenic differentiation of osteoblast.^42^ Similarly, Zfp521 depressed the osteoblast differentiation, skeletal development, and bone homeostasis via attenuation of Runx2 activity by targeting HDAC3 in mice.^43^ miR‐193b‐3p and miR‐188 mediates the chondrogenesis, adipogenesis and osteogenesis of BMSCs by directly targets HDAC3.^26^, ^28^ It is worth noting that HDAC3 mediated physiology and behavioural mal‐adaptions of alcohol dependence in rat^44^; HDAC3 was involved in the chronic alcohol‐binge mediated liver injury.^45^ In our study, in vitro, the results showed that alcohol obviously upregulated the gene and protein levels of HDAC3 in BMSCs. However, the antagonist of Nex reversed the alcohol‐induced inhibitory effects on osteogenic‐associated markers. Moreover, Nex also largely abolished the inhibition of alcohol on mineralization node formation and ALP activity of BMSCs. In vivo, alcohol feeding dramatically increased the level of HDAC3 in the osteopenia model of mice, and agomir‐4286 distinctly downregulated HDAC3 in mice treated with alcohol. Taken together, these data indicated that alcohol inhibited the osteogenesis of BMSCs via upregulating HDAC3.

The osteogenic differentiation, bone homeostasis and remodelling need a variety of oxygen, nutrients and minerals, which are delivered by blood vessels.^13^ The crosstalk between angiogenesis and osteogenesis demonstrates that angiogenesis has an important role in the pathogenesis of osteopenia.^46^, ^47^ Our experimental results indicated that alcohol significantly inhibited the proliferation, migration and tube formation of HUVECs, and downregulated the level of angiogenic‐associated markers in HUVECs. Moreover, alcohol consumption remarkably decreased the level of angiogenic associated markers and reduced the vascular density in the distal femur. It is interesting to note that VEGF‐induced angiogenesis is negatively regulated by HDAC3 via regulating the expression of plasminogen activator inhibitor‐1 (PAI‐1).^48^ Moreover, HDAC3 downregulates the invasion, tumorigenic and angiogenic response of cancer cells.^48^ In this study, we found that alcohol significantly upregulated both gene and protein production of HDAC3 in HUVECs. However, Nex could ameliorate the alcohol‐induced inhibition on angiogenic‐associated markers. Moreover, Nex could greatly restore the function of migration and tube formation of alcohol‐treated HUVECs. When taken into account the fact that agomir‐4286 distinctly downregulated the level of HDAC3 in mice, we concluded that alcohol inhibited the angiogenesis in alcohol‐induced osteopenia via upregulating HDAC3.

Taken together, for the first time, we demonstrated that miR‐4286 might function via intimate osteogenesis‐angiogenesis pathway to alleviate alcohol‐induced osteopenia through the regulation of HDAC3. Hence, miR‐4286 may be a potential diagnostic and therapeutic target for alcohol‐induced bone loss.

## CONFLICT OF INTEREST

The authors declare no competing interests existed.

## AUTHOR CONTRIBUTIONS

HY and KW contributed equally to this work. YG and HY designed the study. HY, KW, PL, PL, DZ, JY, QY, YH, JG and YC conducted the experiments. HY, KW, YC and YG acquired the data. HY, KW, ZA, YC and YG analysed the data. HY, KW and YG wrote the manuscript.

## Supporting information

Table S1

## Data Availability

The data sets used in this study are available from corresponding authors on a reasonable request.
