# Supplementary material for: miR‐4286 functions in osteogenesis and angiogenesis via targeting histone deacetylase 3 and alleviates alcohol‐induced bone loss in mice
Source: Cell Prolif. 2021 May 10;54(6):e13054. doi: 10.1111/cpr.13054 (PMC8168416; doi:10.1111/cpr.13054)
Supplement: Supplementary file 1 — Table S1 [file CPR-54-e13054-s001.docx]

**Table S1**. The RT-PCR primers used in this study.

| Gene | Forward primers | Reverse primers |
| --- | --- | --- |
| mus-GAPDH | 5’-CAGGTTGTCTCCTGCGACTT-3’ | 5’-TATGGGGGTCTGGGATGGAA-3’ |
| mus-COL I | 5’-CTCAAGAAGTCCCTGCTCCTC-3’ | 5’-GACTGTCTTGCCCCAAGTTC-3’ |
| mus-OCN | 5’-GCATCCTTGGCTTTGCAGTC-3’ | 5’-AGTGTTTGCTGTAATGCGCC-3’ |
| mus-OPN | 5’-CCGTTTAGGGCATGTGTTGC-3’ | 5’-CCGTCCATACTTTCGAGGCA-3’ |
| mus-HDAC3 | 5’-TCCCGAGGAGAACTACAGCA-3’ | 5’-CAGGCCGTGAGAGTTTGAGG-3’ |
| has-VEGF | 5’-CTGGGCTGTTCTCGCTT-3’ | 5’-CCCCTCTCCTCTTCCTTCT-3’ |
| has-PDGF | 5’-AGAGGACACGGGAAGGC-3’ | 5’-TGGCTGCTTTAGGTGGGT-3’ |
| has-EGF | 5’-TCAGTATGATACCTCACCCAGC-3’ | 5’-GGGTAGCCGTGTTCTCATGT-3’ |
| has-GAPDH | 5’-CCTTCCGTGTCCCCACT-3’ | 5’-GCCTGCTTCACCACCTTC-3’ |
| has-OPN | 5’-CTCCATTGACTCGAACGAC-3’ | 5’-GTGAAAACTTCGGTTGCTG-3’ |
| has-COL1 | 5’-GACATCCCACCAATCACCTG-3’ | 5’-CGTCATCGCACAACACCTT-3’ |
| has-OCN | 5’-AGCCTTTGTGTCCAAGCA-3’ | 5’-CCAGCCATTGATACAGGTAG-3’ |
| has-HDAC3 | 5’-GAGCAGGGACTTCAGCCTAC-3’ | 5’-GGGATTGTGTGAACGCCAAC-3’ |
| miR‐4286 | 5’-ACCCCACUCCUGGUACC-3’ | 5’-UACCAGGAGUGGGGUUU-3’ |
| U6 | 5’-CTCGCTTCGGCAGCACA-3’ | 5’-AACGCTTCACGAATTTGCGT-3’ |
